# Setting Characteristics, Mechanical Properties and Microstructure of Cement Pastes Containing Accelerators Mixed with Superabsorbent Polymers (SAPs): An NMR Study Combined with Additional Methods

**DOI:** 10.3390/ma12020315

**Published:** 2019-01-20

**Authors:** Yanliang Ji, Zhenping Sun, Chao Chen, Leo Pel, Ahmed Barakat

**Affiliations:** 1 Key Laboratory of Advanced Civil Engineering Materials, Ministry of Education, Tongji University, Shanghai 201804, China; chenchao1254255@163.com; 2 School of Materials Science and Engineering, Tongji University, Shanghai 201804, China; 3 Eindhoven University of Technology, Department of Applied Physics, Transport in Permeable Media, P.O. Box 513, 5600 MB Eindhoven, the Netherlands; l.pel@tue.nl (L.P.); a.barakat@tue.nl (A.B.)

**Keywords:** cement, accelerators, superabsorbent polymers, NMR relaxometry, water distribution

## Abstract

In this study, the setting property and mechanical strength of cement pastes containing accelerators (CPCA) with or without superabsorbent polymers (SAPs) were first studied. The early microstructure evolution and water distribution at 7 and 28 days were probed by 1D (*T*_2_) and 2D (*T*_1_-*T*_2_ maps) H^1^ nuclear magnetic resonance (NMR) relaxometry, and the microstructure was systematically investigated by using mercury intrusion porosimetry (MIP), back-scattered electron (BSE) image and energy-dispersive X-ray spectroscopy (EDX) analysis. Results showed that the SAPs in the cement paste containing accelerators had various influences on setting time and compressive strength depending on the type of accelerators. The presence of SAPs in the cement paste containing alkaline free accelerators could alleviate the decrease of internal relative humidity, promote hydration and help to modify the pore structure. Moreover, it was observed that the SAP cavities could be nicely filled with calcium hydroxide (CH) in the cement paste with alkaline free accelerators.

## 1. Introduction

Sprayed concrete is a kind of commonly used material, which can form homogeneous structures with proper thickness, as well as sufficient rigidity and strength in a very short time. The design of sprayed concrete involves the components of cement paste containing accelerators (CPCA), densely packed aggregates (size generally below 16 mm) and an appropriate water to cement ratio (this depends on the demanding strength) [1,2]. A rapid setting CPCA is widely regarded as the essential element, which makes it possible for the use of the sprayed cement-based materials.

To improve properties of CPCA, i.e., viscosity or setting time, a high dosage of accelerators and high content of binder materials are always used in practice. However, the high dosage of an accelerator may have negative impacts on long-term mechanical properties and durability [2,3,4]. The high content of binder materials will not only result in a high cost and difficulties in manufacture but also increases the autogenous shrinkage of sprayed concrete [2,3]. Thus, other ways to further improve the properties of the sprayed concrete needs to be considered. As proposed in Snashall’s [5] and Jensen’s patents [6], the use of superabsorbent polymers (SAPs) will increase viscosity and decrease the rebound of wet-mix sprayed concrete. It was observed that the uptake of water by SAPs created a change in viscosity during placing and allowed the build-up of thick layers [7]. The use of SAPs provides a way to improve the performance of sprayed cement-based materials.

However, for the lacking systematic study, three challenging issues may remain in the application of SAPs on sprayed concrete. First, the influence of SAPs on the microstructure of the cement paste is not fully understood. Without adding extra water, the addition of SAPs can reduce the local water to cement ratio by absorbing water internally [8]. However, a fraction of water may be inhibited by the polymer films, which then reduce the hydration degree of the cement paste. Second, the surroundings of the water containing SAPs particles are supposed to exist in different chemical and physical conditions resulting from accelerated cement hydration [9]. This may lead to various effects on the water-releasing process and therefore alter the effects of the SAPs on the structure. Third, the SAPs can generate defects [9,10] in cement-based materials due to their hollow structure. Thus, whether the hydrates involved in the accelerated hydration process can fill up defects and compensate for the negative influences, is the third issue.

For a better application of SAPs on cement paste containing accelerators, further experimental investigations on the influence of SAPs on the properties and microstructure of cement paste containing accelerator are needed. In this study, the setting time and compressive strength development of cement paste containing accelerators with or without SAPs were first investigated. In order to study the water-releasing processes of SAPs and microstructure variation, proton nuclear magnetic resonance (^1^H NMR) technique was employed to extract 1D NMR data from the cement paste containing accelerator during a hydration time of 3 days. With the 2D *T*_1_-*T*_2_ maps, the water distribution of cement paste containing accelerator with or without SAPs, cured for 7 and 28 days, were compared and discussed. The pore structure was investigated by mercury intrusion porosimetry (MIP). Furthermore, the hydrate filling, which occurred in the SAPs cavities and the curing effect were studied by SEM imaging in back-scattered electron (BSE) mode and EDX analysis.

## 2. Materials and Experiment

### 2.1. Materials

White Portland cement with a ferric content of 0.28% was used, and its Blaine specific surface area was 396 m^2^/kg. The detailed chemical composition (determined by a X-ray Fluorescence Spectrometer, BRUKER AXS, Karlsruhe, Germany) and physical properties (provided by the manufacturer: Onoda, Nanjing, China) of cement are summarized in Table 1. Two types of typical accelerators were selected, the alkaline accelerator (A1; Al(OH)_3_ content: 37.62%, NaOH content: 12.12%; pH = 12.6, and alkaline free accelerator (A2, Al_2_(SO_4_)_3_ content: 51.2%, pH = 2.4, Gao Qiang Co., Ltd., Linyi, China). The added content dosage by cement weight was 4.0% and 7.0%, respectively. Solution-polymerized, sodium polyacrylate-based and cross-linking SAPs (a type of H-100, manufactured by Sen Fei Chemical Corporation, Shanghai, China) with irregular particle shape were used. Its average size was 112.2 μm, and maximum size was 160 μm. The particle density and water absorption capacity was 0.72 ± 0.02 g/mL and 230 ± 2 g/g, respectively. Deionized water was used during all the sample preparations and measurements.

The preparation of the samples of cement paste containing accelerators (CPCA) was based on the method of JGJ/T-477 [11]. The cement (premixed with or without SAPs) was mixed with water for 30 s at a low speed and then the mixer was turned off for 60 s (providing enough time for the water absorption of the SAPs). After that, the weighed accelerator was rapidly injected by syringe, followed by remixing for 5 s at a low speed, and 15 s at a high mixing speed by a standard mixer. Mix design of the cement paste and mortar in this study is shown in Table 2. The CPCA mortars with a sand-cement ratio of 1.5 were prepared for the mechanical strength, in which the standard siliceous sands (maximum size was 4.75 mm) were used in this study (according to Table 2). In this study, the microstructural characterizations were all carried out on cement pastes with the same w/c ratio of 0.45. It was necessary in order to quantify the effect of the SAPs on the microstructural development and phase composition. The setting time of the sample was measured by using Vicat’s Apparatus (SHEN RUI Co., Ltd., Shanghai, China), and the compressive strength of the mortars (40 mm × 40 mm × 160 mm) was tested at curing times of 1, 3, 7 and 28 days according to the standard methods [11,12]. Three parallel samples were prepared to determine each value for the compressive strength. It should be noted that except the compressive strength test for the mortar samples, all the tests or measurements in this study were conducted on the cement paste sample.

### 2.2. Nuclear Magnetic Resonance Tests

As a nondestructive and noninvasive method, NMR not only can help to identify microstructure variations but also provides important insights into the water migration between the SAPs and cement paste [13]. The NMR instrument (MICRO-MR20; Niumag Electric Corporation, Shanghai, China) used for this study has a magnetic field of 0.5 T and a 25 mm Radiofrequency (RF) coil. After mixing, the sample was immediately transferred (approximately 6.5 g) into a glass tube, which was then sealed by a polytetrafluoroethylene (PTFE) film.

#### 2.2.1. Transverse Relaxation Time

The transverse relaxation time (*T*_2_) was measured using (Carr-Purcell-Meiboom-Gill) CPMG sequence and the parameters including the echo time (*τ*_1_ = 0.15 ms) and the number of scans (*NS* = 8) were kept constant throughout the 1D NMR experiment. The *T*_2_ relaxation curve was fitted to a multi-exponential curve by using the inverse Laplace transform algorithm [13,14].

#### 2.2.2. Two-Dimensional *T_1_*-*T_2_* Maps

**Figure 1:**
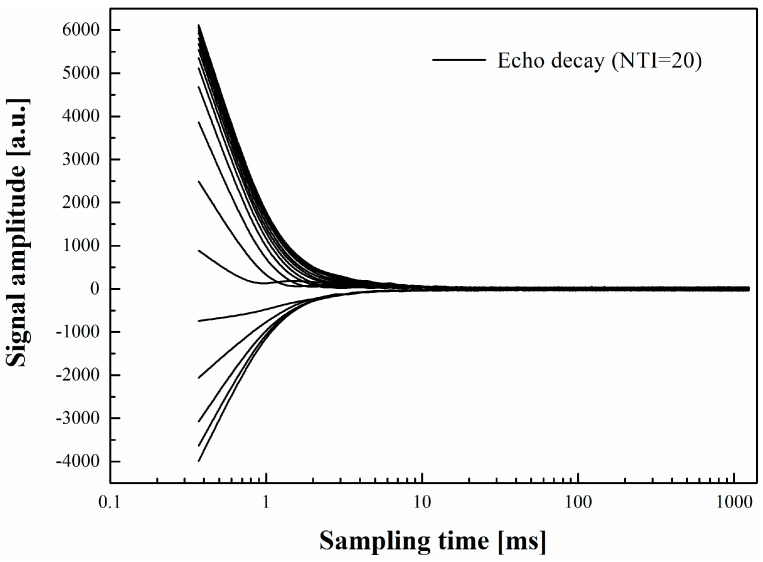
A representative decay of the proton magnetization curves (the sample of CPCA2-SAPs at 7 days) as measured by nuclear magnetic resonance at 0.5 T.

For measuring the microstructure and identifying the water in SAPs or pores in the late stage, the *T*_1_-*T*_2_ maps of the sample were used. The fixed parameter settings of the NMR equipment including echo time (*τ*_1_ = 0.15 ms), number of scans (*NS* = 32) and the number of invert time (*NTI* = 20) for the measurement were selected. The 2D *T*_1_-*T*_2_ exponential decay data was obtained using IR-CPMG sequence (IR is short for the inversion recovery) [15,16]. The sampling data can be expressed as a Fredholm integral formula:

(1) $$M(\tau_{2},\tau_{1})=M_{0}\iint \left(1-2\exp^{-\frac{\tau_{1}}{T_{1}}}\right)\exp^{-\frac{\tau_{2}}{T_{2}}}S(T_{2},T_{1})dT_{1}T_{2}$$

where *τ*_2_ is the recovery time, *τ*_1_ is the echo time and *M*(*τ*_2,_ *τ*_1_) is the corresponding magnetization signal. *M*_0_ is the initial magnetization signal. *S*(*T*_2_, *T*_1_) corresponds to the probability density of molecules with *T*_1_ and *T*_2_. Representative decay curves of the sample are depicted in Figure 1. The 2D *T*_1_-*T*_2_ maps were then obtained with the inversion algorithm based on the Tikhonov normalization method [15,16,17].

### 2.3. Mercury Intrusion Porosimetry

The mercury intrusion porosimetry (MIP) equipment (Poremaster GT-60; QUANTACHROME INS Corporation, Nanjing, China) was utilized to characterize the pore structure of samples. After 7 days and 28 days of curing, the crushed sample was immersed in ethyl alcohol for two days to terminate hydration, and then the samples were dried at 44 °C (to avoid ettringite phase decomposition) until a constant mass was attained. The samples were then crushed into maximum particle size of 5.0 mm and the total mass of the sample for the MIP measurement was around 1.5 g.

### 2.4. Back-Scattered Electron Imaging and EDX Analysis

The microstructure of the samples in this study was investigated with the back-scattered electron (BSE) mode using an environmental scanning electron microscope (SEM, Quanta 200 FEG, FEI Co., Ltd., Hillsboro, OR, USA), which was equipped with a field emission gun in high vacuum (a voltage of 20 kV). The dried samples were impregnated with low viscosity epoxy resin under a high vacuum condition (about 0.08 MPa). After a series of grinding and polishing procedures (the detailed procedures were described in the literature [18]), the polished sections of the samples were coated with a thin film of carbon (about 5 nm) for the test.

**Figure 2:**
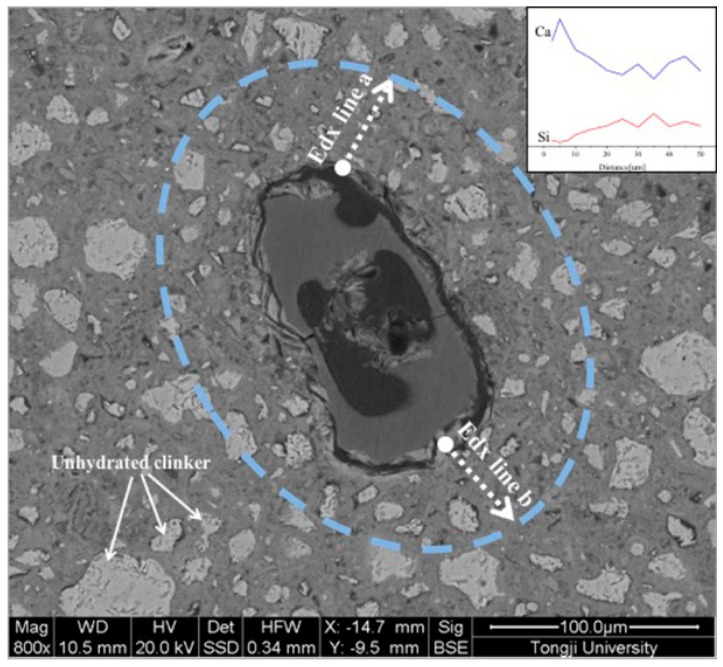
EDX line analysis of CPCA2-SAPs (the insert is the spectrum of EDX line referring to the corresponding element concentration).

The different clustered phases of the polished sections were further investigated with energy dispersive spectrometry (EDX, FEI Co., Ltd., Hillsboro, OR, USA) analysis. The EDX line analysis was performed on the SAPs containing samples to observe the microstructure around the SAPs and composition of the filling products in the SAPs voids due to cement hydration. Ten measurements were performed on different SAPs particles in sample and Ca/Si ratios were calculated and plotted against the distance away from the rim of the SAPs. Figure 2 presents an example of EDX line measurement on the CPCA2-SAPs sample. During the measurement, the line analysis was placed in the regions with less unhydrated clinkers (brighter zone) existed in the images.

## 3. Results and Analysis

### 3.1. Setting Time

**Figure 3:**
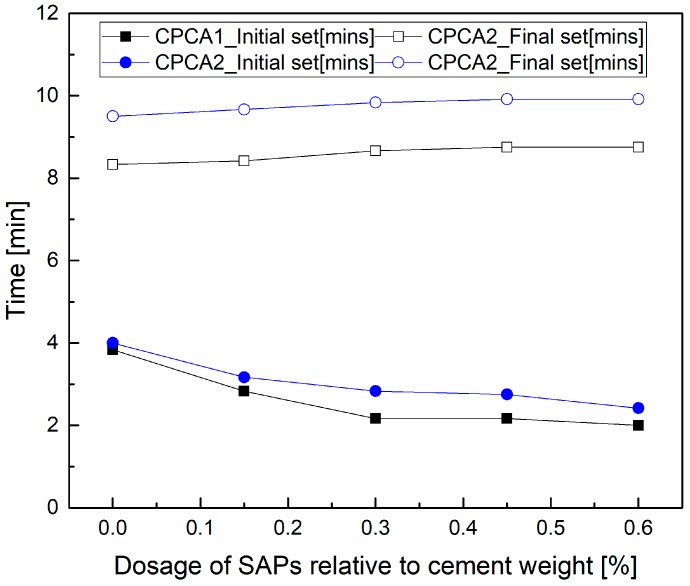
The setting time for the cement paste containing accelerators as a function of the dosage of SAPs.

Regarding the importance of the setting properties for practical applications of sprayed concrete or mortar, the influence of SAPs dosages on the setting time of the cement paste containing alkali (CPCA1) or alkaline free accelerators (CPCA2) was first investigated. In Figure 3, it can be seen that the increased dosage of SAPs in the sample could shorten the initial setting times of the accelerator containing cement pastes while the final setting time was slightly extended. With the SAPs dosages increasing to 0.30%, the initial setting time of the cement paste with the alkaline accelerator and the alkali-free accelerator could be reduced to 40.47% and 29.17%, respectively. Although the use of SAPs retards the final setting time, the values of the final setting time obtained were within the acceptable range of standards (the setting times were all less than 10 min). The hypothesis for the trend of setting times is that the SAPs can absorb water inside their body, which results in a denser structure in a few minutes. Thus, the initial setting time can be shortened. Later on, the hydration extent of the cement with SAPs were considered as lower than the sample without SAPs. Additionally, the SAPs in the cement paste may also weaken the local structure. Therefore, the longer setting time could be found.

### 3.2. Compressive Strength

**Figure 4:**
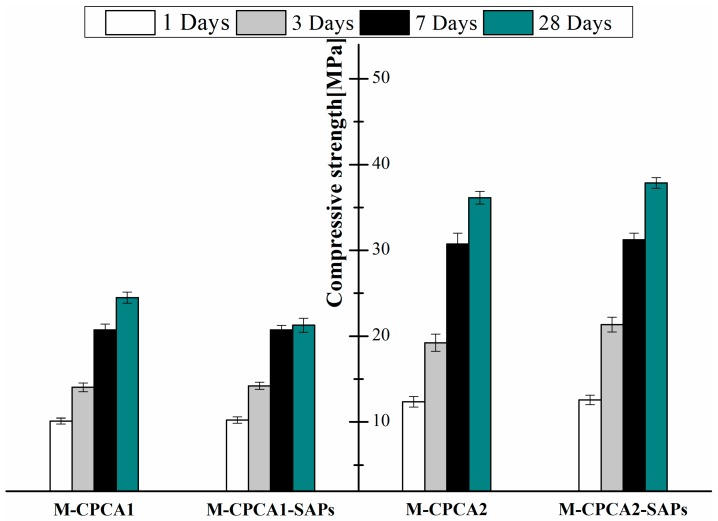
Compressive strength of mortar made by cement paste containing accelerator with or without SAPs (The error bar is the standard deviation of the compressive strength).

The compressive strength of the samples with or without SAPs at 1, 3, 7 and 28 days were depicted in Figure 4. As can be seen, there was a noticeable increase in compressive strength as a function of increasing curing time. For the alkali accelerated system, the sample with SAPs had higher compressive strength at 1 and 3 days but a lower compressive strength at 28 days in comparison with the cement paste containing alkaline accelerator (a reduction of about 3.21 MPa due to the addition of SAPs at 28 days). Additionally, the CPCA2-SAPs sample had a higher compressive strength in comparison with CPCA2 mortar without SAPs at 1, 3, 7 and 28 days of curing time. The addition of SAPs led to an increase of 4.76% in compressive strength of the sample when compared to CPCA2 at a hydration time of 28 days.

The experimental results above indicated that the presence of SAPs in cement paste could lead to various influences on both the setting time and mechanical strength when different types of accelerators were selected. The influencing mechanism of the SAPs on the properties of the cement paste containing accelerator is complicated because it may involve the local w/c ratio reducing effect [7], water curing effect [8] or uncertain impacts of the defects originating from the SAPs [10]. Thus, to fully understand these effects, further investigations on the microstructure development of the cement paste containing accelerator with or without SAPs should be extended.

### 3.3. Evolution of the Microstructure

**Figure 5:**
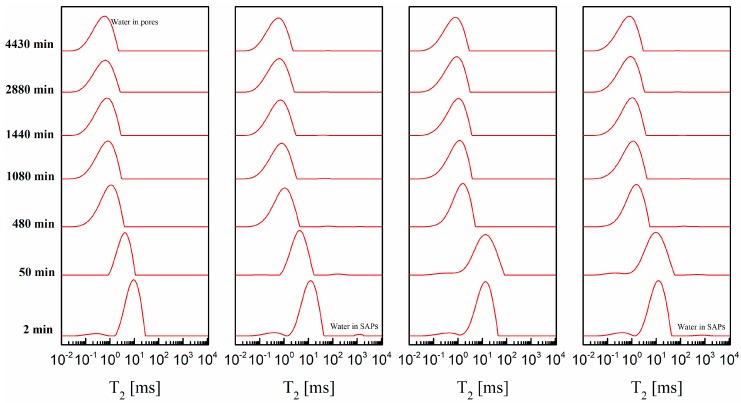
Selected relaxation time distributions of the cement paste containing accelerators with or without superabsorbent polymers (SAPs).

Figure 5 shows the transverse relaxation time (*T*_2_) distribution of cement paste containing accelerator with or without SAPs within 4430 min. The relaxation time of the water inside the sample without SAPs are represented by a broad peak, which correlates with the water in the pores of the cement paste and a tiny peak in the *T*_2_ range of 0.10–1.0 ms (originating from the water in the flocculated structure [14]). The water inside the SAPs could be observed for CPCA1-SAPs and CPCA2-SAPs samples in the *T*_2_ range of 100–1000 ms. This observation is consistent with the findings in the work of Nestle et al. [13] which studied the water distribution and pore structure development in cementitious materials with SAPs by NMR.

In order to investigate the microstructure development beginning at the very early stage (after mixing with water for 2 min), the data collecting time was shortened as much as possible by adjusting the NMR parameters. It would inevitably reduce the relative intensity of the signals of water in the SAPs compared with intensity originating from the water in the pores. Therefore, very small signals were found for the SAPs in Figure 5. It should also be noted that the transforming simulated data was added to achieve the *T*_2_ component below the echo time.

**Figure 6:**
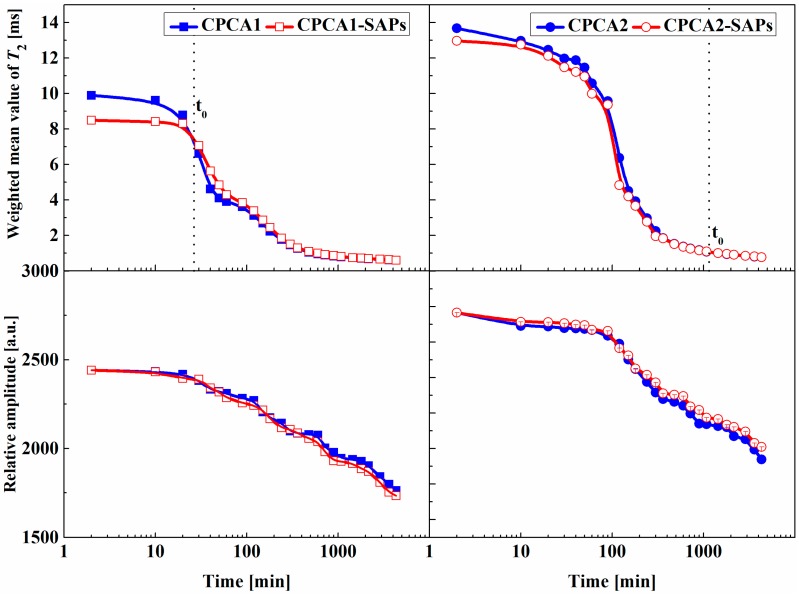
Evolution of weighted mean value of *T*_2_ and relative amplitude of the samples from a hydration time of 2 min to 4430 min.

The microstructure development and water content of the CPCA were investigated through analyzing NMR results including the weighted mean value of *T*_2_ (for the definition see Equation (2)) and the relative amplitude. The results are depicted in Figure 6.

(2) $$T_{2,w}=\sum_{T_{2,min}}^{T_{2,\max}}\left((T_{2,i})\cdot \frac{A_{i}}{A_{\mathrm{total}}}\right)$$

where, *T_2_*_,*w*_ is the weighted mean value, *T_2_*_,*i*_ is the transverse relaxation time at measurement *i*, *A_i_* is the relative amplitude for the measurement *i* and *A_total_* is the total amplitude of the *T*_2_ peak.

The evolution of the weighted mean value of the transverse relaxation time (*T*_2_) and the relative amplitude of the cement paste containing accelerators with or without SAPs is shown in Figure 6. As can be seen, both the weighted mean value of *T*_2_ and the relative amplitude of all the tested samples decreased as a function of time. It was also found that the CPCA2-SAPs could maintain a higher relative amplitude than that of the CPCA2. This observation suggests that the water consumption in CPCA1-SAPs is higher than CPCA1.

Before the time t_0_ (CPCA1-SAPs: t_0_ = 30 min and CPCA2-SAPs: t_0_ = 1440 min), the smaller *T*_2_ value could be observable due to a higher volume/surface ratio (*T*_2_ is proportional to the pore size [13,14]) resulting from the lower effective w/c ratio in the paste with SAPs. The smaller *T*_2_ value of the CACP1-SAPs and CACP2-SAPs in the first few minutes could account for the shortened initial setting time and higher strength at 1 and 3 days. After the time t_0_, it was found that the *T*_2_ values of the CPCA1-SAPs and CPCA2-SAPs were larger than that of the sample without SAPs. In the conceptual model that Gajewicz et al. proposed [19], water molecules were believed to preferentially fill the smaller pores rather than the larger pores because of the capillary action due to drying of the cement paste. The larger *T*_2_ value may be caused by the release of water from the SAPs to pores compared to the SAPs-free reference samples.

### 3.4. 2D T_1_-T_2_ Maps

**Figure 7:**
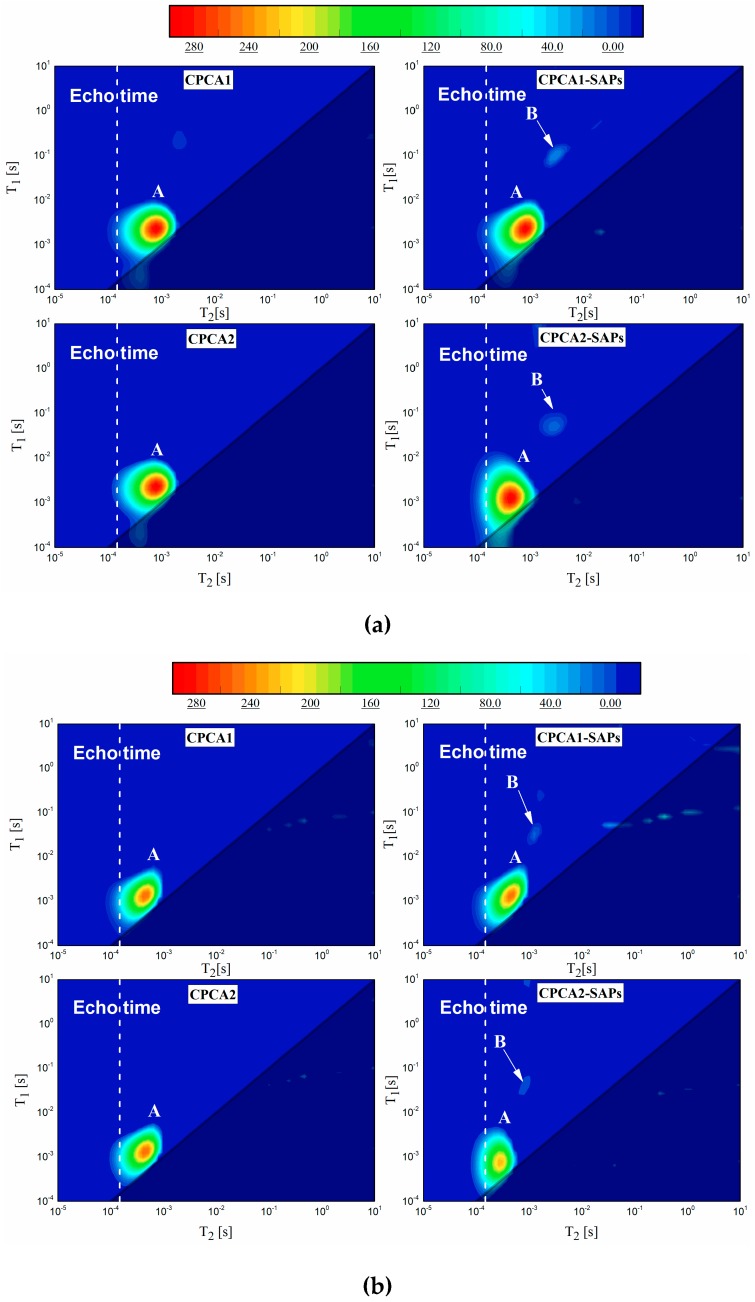
*T*_1_-*T*_2_ maps of the cement paste containing accelerator sample with or without SAPs at (**a**) 7 and (**b**) 28 days (The line indicates *T*_1_/*T*_2_ = 1 and the vertical dashed line shows the minimum echo time of the instrument).

*T*_1_-*T*_2_ maps of the cement paste containing accelerator with or without SAPs after aging for 7 (Figure 7a) days and 28 (Figure 7b) days are shown in Figure 7. It can be seen that all the samples had a majority of hydrogen proton signals (region A) around the diagonal in the *T*_2_ range between 10^−4^ s and 10^−3^ s due to the capillary pores in the sample. The organic SAPs could alter the interaction between the water molecules and SAPs internal surface which led to a higher *T*_1_ and *T*_2_. Thus, the remnant water remaining in the SAPs (located at region B in both cement pastes with SAPs in Figure 7) could be distinguished from the water in the capillary pore. The signals located under the line in the maps (*T*_1_/*T*_2_ = 1) were believed to originate from the inversion errors.

Additionally, the signals of each area found in the *T*_1_-*T*_2_ map at 28 days were less than that of the sample tested at 7 days because of the continuing hydration. Figure 7 also indicated region A of the CPCA1 in the *T*_1_-*T*_2_ map had similar coordinate positions compared to that of the CPCA1-SAPs at 7 and 28 days. In comparison with the CPCA2, the kernel of the region A in the CPCA2-SAPs map was found to have shifted from one coordinate (*T*_1_ = 1.55 ms, *T*_2_ = 0.52 ms) to the other coordinate (*T*_1_ = 0.42 ms, *T*_2_ = 0.27 ms) in Figure 7a. The appearance of region A with the extension to the limit of the *T*_1_ resolution (10^−4^ s) was also found for the CPCA2-SAPs in Figure 7. These results suggest that the SAPs in cement paste containing alkaline free accelerator may contribute to forming a denser pore structure and reducing the internal relative humidity decrease. The kernels of region B found in both CPCA1-SAPs and CPCA2-SAPs, as shown in Figure 7b all shifted to a smaller *T*_1_ and *T*_2_ in comparison with the Figure 7a. There are two possible reasons for the variation of region B from 7 days to 28 days of aging. First, the cement hydration may lead to the self-desiccation of the surrounding of SAPs, followed by the consumption of water inside the SAPs. The second hypothetical reason is that the void of the SAPs may be filled up by hydrates generated from 7 days to 28 days of curing aging. The process of the cement hydration is a process that is aimed at minimizing the free energy of the whole system. Thus, the growth of the hydrates in the first place occurs in places where local hydration pressure will be as low as possible. Emptiness in the matrix are such places, whether it is air pore or void of the SAPs and cement hydration products will fill up [20,21].

### 3.5. Pore Structure

**Figure 8:**
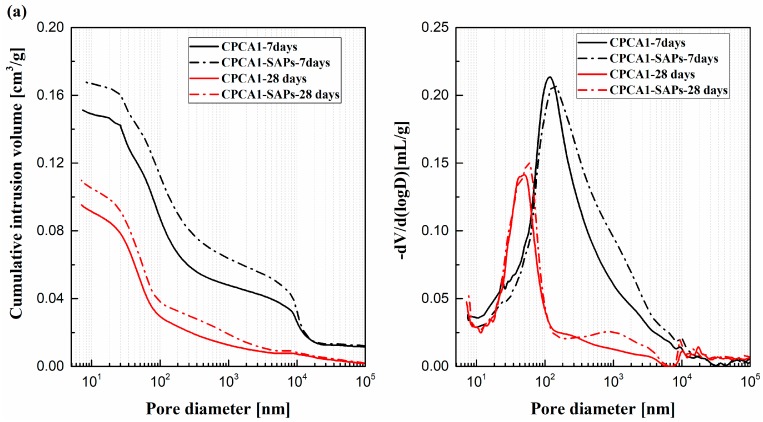

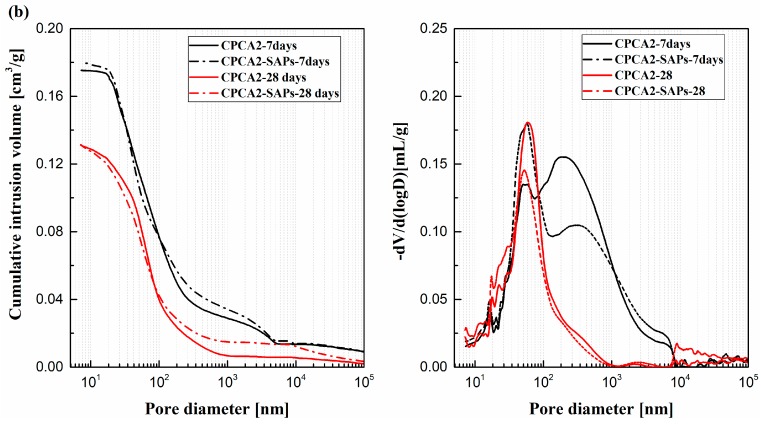
Cumulative intrusion pore volume and pore size distribution of the sample with or without SAPs (**a**) cement paste mixed with alkali accelerator; (**b**) cement paste mixed with alkaline free accelerator.

The cumulative intrusion pore volume and pore size distribution of the accelerator-containing cement paste with or without SAPs are shown in Figure 8. It can be observed that the total porosity and the critical pore diameters of all samples were decreasing with increasing hydration time from 7 days to 28 days of curing. Figure 8a indicates that the CPCA1 containing SAPs had a higher porosity (higher cumulative Hg intrusion) and the most probable pore size at 7 and 28 days were also larger compared with the sample without SAPs. The MIP results were also consistent with the NMR analysis of *T*_1_-*T*_2_ maps.

The porosity of the CPCA2-SAPs was slightly higher (but the curves of cumulative intrusion of Hg were very similar) than that of the sample lacking SAPs at 7 and 28 days of curing, as shown in Figure 8b. Presumably, the ink-bottle-like pores created by the SAPs may have increased the porosity of the sample [22]. The pore size distribution of the CPCA2-SAPs was found to have shifted to a smaller pore size range in contrast with the CPCA2. It was also found that the SAPs could decrease the pore volume in the range of 100–1000 nm but increase it when the pore size was smaller than 10 nm at 7 days of curing. This observation was attributed to the presence of SAPs in the cement paste, yielding to a lower w/c ratio, where the pore structure modifications resulting from the addition of SAPs may also be due to the curing effect of the released water from the SAPs (in agreement with the analysis of region A in the *T*_1_-*T*_2_ maps) [23].

### 3.6. Back-Scattered Electron and EDX Analysis

**Figure 9:**
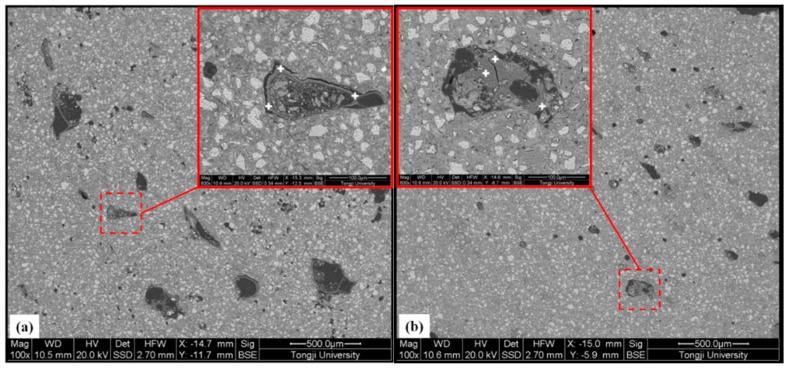
Back-scattered electron images of the CPCA1 with SAPs (**a**) and CPCA2 with SAPs (**b**) at a hydration time of 28 days. The inserting images are local amplification (800×) of the typical SAPs particles found in the sample.

BSE images from polished samples of the CPCA1 with SAPs and CPCA2 with SAPs at 28 days of curing are given in Figure 9. It can be seen from the images that the voids in the SAPs were large in the alkaline accelerator-containing sample even after 28 days of curing. In the cement paste with alkali-free accelerators, the original SAP reservoirs were partially filled with hydration products. This finding favors the results of the *T*_1_-*T*_2_ maps in Figure 7 that the signals of zone B (also smaller relaxation time) were lower in CPCA2-SAPs in comparison to the CPCA1-SAPs. 

From the results of the EDX point measurement at the corresponding location as shown in Figure 9, the mass fraction estimations for the CPCA1 with SAPs were: Ca: 55.64% ± 0.4%, O: 24.28% ± 3%, Na: 7.59% ± 0.4%, (Al, Si, K): 12.49% ± 2%. Based on the results and grey value (contrasted with the known phase such as pores and unhydrated cement grains), it was confirmed that the constitution of the rim of the SAPs consisted of a certain amount of calcium hydroxide (CH) which can grow along with the rim. For the CPCA2-SAPs sample, the EDX measurements (Ca: 36.84% ± 1%; O: 60.50% ± 2%) suggested that relatively pure and integral CH were formed in SAP reservoirs in the cement paste containing alkali-free accelerators. It has been demonstrated in the literature [13,24] that ${\mathrm{SO}}_{4}^{2-}$ can inhibit the CH yield in the crystal face 001, and may result in the formation of CH with a hexagonal-platelet morphology. Thus, with the addition of the alkali-free accelerator (aluminum sulfate, 51.2%), the voids created by the SAPs can be adequately filled by the CH. In the later hydration stage (during 7 to 28 days), the existence of the SAPs cavities may offer small spaces for the hydrates to grow, which is beneficial for the hydration of the accelerator-containing cement. Therefore, hydrates could be found in the SAPs reservoirs in Figure 9.

**Figure 10:**
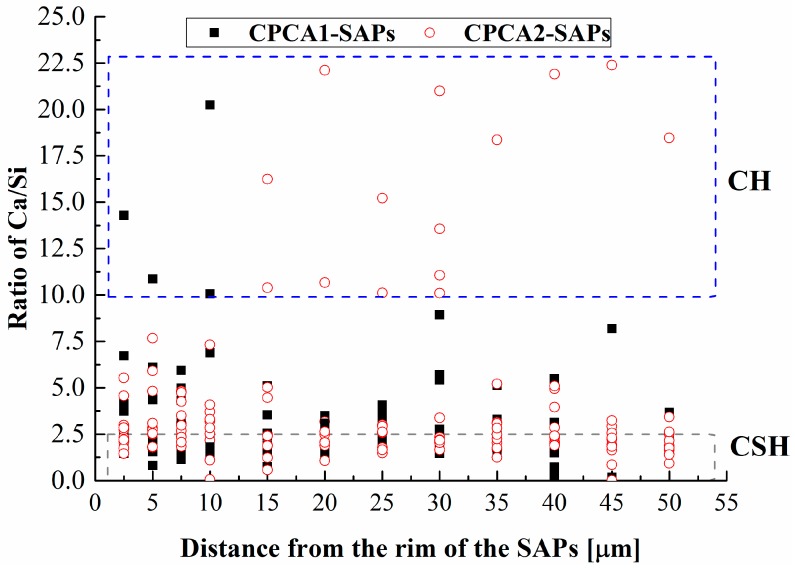
Ca/Si molar ratio as a function of the distance from the rim of the superabsorbent polymers (SAPs) as determined by the EDX.

The water curing effect of the SAPs on the chemical composition around the SAPs reservoirs (2.5–50 μm) was investigated by the EDX method, and the resulting Ca/Si ratio as a function of the distance from the rim of the SAPs is shown in Figure 10. Within a distance of 15 μm, more data on the Ca/Si ratio for values lower than 2.5 can be found in Figure 10. This observation implies that the surrounding of the SAPs tended to generate calcium-silicate-hydrate (C-S-H) gels [18,25], and more C-S-H gels may exist around the SAPs in the sample of CPCA1-SAPs in comparison with the CPCA2-SAPs. Moreover, a large amount of data on the Ca/Si ratio up to values of 10 can be found in the CPCA2-SAPs for distances ranging from 15 to 50 μm. It indicates that the range of the curing effect due to the SAPs in the cement paste with alkali-free accelerators is larger than that of the SAPs in the cement paste containing alkaline accelerator.

## 4. Conclusions

Based on the analyses of the results, the conclusions resulting from this study are summarized as follows:

(1) The addition of SAPs could reduce the initial setting time of cement pastes containing accelerators. In comparison with the SAPs-free samples, the cement paste containing alkali accelerators with SAPs has a lower compressive strength in the later stage, while the CPCA2-SAPs sample has a higher compressive strength during 28 days.
(2) The smaller *T*_2_ value implies a denser structure is formed which accounts for the shortened initial setting time. Compared with the SAP-free samples, the SAPs could decrease the total relative amplitude of the cement paste containing an alkali accelerator but increase it when the alkaline-free accelerator was selected.
(3) *T*_1_-*T*_2_ maps indicated that measurable water was composed of two fractions discerned as water in pores and water in SAPs. The extension of water in pores to the limit of the *T*_1_ resolution (10^−4^ s) which suggested that SAPs in cement paste containing alkaline-free accelerator helps to form a denser structure.
(4) The cement paste containing accelerator with SAPs had a higher porosity compared to the sample lacking SAPs. For the alkaline-free accelerator cement paste. The peak in the pore size distribution of the sample with SAPs was also shifted to smaller pore size ranges, in contrast with the SAPs-free samples.
(5) The voids of the SAPs are large in the alkaline-accelerator containing cement pastes, and in the alkali-free accelerator containing sample, the original SAP reservoirs are filled with CH. The EDX results of Ca/Si ratio indicated that the range of the curing effect caused by the SAPs in the cement paste with alkali-free accelerator was larger than that of the SAPs in the alkali accelerator containing cement paste.

## Figures and Tables

**Table 1:** Chemical composition wt % and physical properties of cement.

	SiO_2_	Al_2_O_3_	Fe_2_O_3_	CaO	MgO	SO_3_	K_2_O	Specific Weight (g/cm^3^)	Blaine Specific Surface Area (m^2^/kg)	Initial Setting Time (min)
Cement	22.9%	2.47%	0.28%	64.6%	2.82%	3.20%	0.44%	3.08	396	105

**Table 2:** Mix design of the cement paste and mortar in this study.

Sample Names	Cement	Water	Sands	Alkaline Accelerator (A1)	Alkaline-Free Accelerator (A2)	SAPs
CPCA1	1	0.45	-	0.04	-	-
CPCA1-SAPs	1	0.45	-	0.04	-	0.0030
CPCA2	1	0.45	-	-	0.07	-
CPCA2-SAPs	1	0.45	-	-	0.07	0.0030
M-CPCA1	1	0.45	1.5	0.04	-	-
M-CPCA1-SAPs	1	0.45	1.5	0.04	-	0.0030
M-CPCA2	1	0.45	1.5	-	0.07	-
M-CPCA2-SAPs	1	0.45	1.5	-	0.07	0.0030
